# Modelling Degradation and Replication Kinetics of the Zika Virus In Vitro Infection

**DOI:** 10.3390/v12050547

**Published:** 2020-05-15

**Authors:** Veronika Bernhauerová, Veronica V. Rezelj, Marco Vignuzzi

**Affiliations:** 1 Viral Populations and Pathogenesis Unit, Department of Virology, Institut Pasteur, CNRS UMR 3569, F-75015 Paris, France; veronica.rezelj@pasteur.fr; 2 Department of Biophysics and Physical Chemistry, Faculty of Pharmacy, Charles University, Heyrovského 1203, 500 05 Hradec Králové, Czech Republic

**Keywords:** Zika virus, in vitro viral kinetics, viral decay, mathematical model

## Abstract

Mathematical models of in vitro viral kinetics help us understand and quantify the main determinants underlying the virus–host cell interactions. We aimed to provide a numerical characterization of the Zika virus (ZIKV) in vitro infection kinetics, an arthropod-borne emerging virus that has gained public recognition due to its association with microcephaly in newborns. The mathematical model of in vitro viral infection typically assumes that degradation of extracellular infectious virus proceeds in an exponential manner, that is, each viral particle has the same probability of losing infectivity at any given time. We incubated ZIKV stock in the cell culture media and sampled with high frequency for quantification over the course of 96 h. The data showed a delay in the virus degradation in the first 24 h followed by a decline, which could not be captured by the model with exponentially distributed decay time of infectious virus. Thus, we proposed a model, in which inactivation of infectious ZIKV is gamma distributed and fit the model to the temporal measurements of infectious virus remaining in the media. The model was able to reproduce the data well and yielded the decay time of infectious ZIKV to be 40 h. We studied the in vitro ZIKV infection kinetics by conducting cell infection at two distinct multiplicity of infection and measuring viral loads over time. We fit the mathematical model of in vitro viral infection with gamma distributed degradation time of infectious virus to the viral growth data and identified the timespans and rates involved within the ZIKV-host cell interplay. Our mathematical analysis combined with the data provides a well-described example of non-exponential viral decay dynamics and presents numerical characterization of in vitro infection with ZIKV.

## 1. Introduction

Zika virus (ZIKV) is an arthropod-borne virus (arbovirus) primarily transmitted through a bite of infected *Aedes* mosquitoes, belonging to the *Flavivirius* genus, which includes also West Nile virus (WNV), Japanese encephalitis virus (JEV), dengue virus (DENV) and yellow fever virus (YFV). Although its discovery in a Ugandan forest dates back to 1947 [1], the first sporadic outbreaks outside Africa were reported in the Asia-Pacific region in 2007 [2] and 2013 [3]. Since then, it rapidly spread to the Western hemisphere in 2016 [4] where it received public attention due to the association of ZIKV infection with newborn microcephaly and other neurological abnormalities [5,6,7,8]. Currently, no approved vaccine or therapeutic treatments exist to specifically target ZIKV infection and its continuous re-emergence poses an important public health threat, especially in developing countries where disease prevention mostly relies on decreasing the number of transmission events through vector-control strategies.

Understanding the rates and time scales of viral degradation is critical as it may play a significant role in designing effective therapeutics and intervention strategies to control or eliminate the virus. Conventionally, the loss of viral infectivity over time is described by a decreasing exponential function of incubation time, which assumes that each virion has the same probability of losing infectivity at any given time. However, experimental and theoretical work has recognized that exponential law is not an exclusive driver of viral degradation, as, for example, heterogeneity in the viral population was proposed to cause a deviation in the shape of the infectious virus degradation curve from the exponential law [9,10]. The in vitro kinetics of *Flavivirus* genus has been mathematically studied in terms of activation of the innate antiviral defense in vitro in DENV [11]. In this study, exponential decay was assumed from the observed data and the estimated time DENV remained infectious was reported to be 2.5 h. Experimental studies reported prolonged structural stability (measured as a loss of infectivity) of ZIKV and reduced stability of DENV during short incubation periods [12]. In [13], stability of the contemporary (H/PF/2013 and Paraiba/2015) and historic (MR766) ZIKV strains were were quantified using the exponential decay model and half-lives of 5.1 h, 5.2 h and 3.5 h for H/PF/2013, MR766, and Paraiba/2015 strains, respectively, were found. In addition, this study directly compared the loss of infectivity in dengue virus type 2 (DENV2) and WNV generated reporter virus particles, whose infectious half-lives were quantified to be 5.2 h and 17.7 h, respectively, which was in agreement with previous findings [14,15,16].

Mathematical models of in vitro viral infections help provide accurate estimations of the rates of the processes affecting virus-cell interactions and time scales on which these processes occur. These measures have been determined for a number of viruses, including HIV-1 and simian–human immunodeficiency virus [17,18,19,20,21,22], hepatitis C virus [23,24,25,26,27], poliovirus [28,29,30], influenza A virus and its various strains [31,32,33,34,35,36,37], West Nile virus [38] and Ebola virus [39,40]. Specifically in [10,20,21,32,36], different aspects of viral replication cycle were considered to provide a comprehensive description of in vitro viral spread. In particular, the decay of infectious viral particles and the integrity of viral RNA were measured over time to determine the rates at which infectivity and integrity are lost. Cellular infection was carried out at different multiplicities of infection (MOI) to capture different dynamics of early virus replication. Viral load was quantified at select times to obtain kinetic profiles. The collected data were used to fit mathematical models of in vitro viral dissemination to estimate parameters descriptive of the viral replication cycle.

In this study, we addressed how ZIKV (MR766 strain) loses infectivity. As in the studies above, we measured the degradation of infectious ZIKV over time and discovered that it seems not to be governed by the exponential law. Rather, infectivity of ZIKV is maintained for the first 24 h after which it begins to decline. The manner with which ZIKV loses infectivity thus presents an example that complements previous observations on ZIKV decay kinetics. We further characterized the degradation of infectious ZIKV, and showed that a model in which viral decay is gamma distributed could accurately describe ZIKV loss of infectivity. We then incorporated this viral decay model into a mathematical model of viral dynamics, that was developed by Beauchemin and colleagues [10,20,21,32,36], to quantify the main determinants of ZIKV infection kinetics in vitro. As in [10,20,21,32,36], we measured temporal changes in the viral load in the extracellular milieu in a series of experiments reflective of different aspects of the viral replication cycle and fit these data to the mathematical model to quantify the model parameters. To minimize the influence of immune responses to ZIKV infection, we used a mammalian cell line (Vero) that is incapable of producing type I interferon in response to viral infections [41,42]. The proposed models reproduced experimental data with high accuracy and delivered, to the best of our knowledge, the first numerical characterization of in vitro ZIKV infection.

## 2. Materials and Methods

### 2.1. Cells

Vero and HEK-293T cells were maintained in Dulbecco’s modified Eagle’s medium (DMEM), supplemented with 10% fetal calf serum (FCS) and 1% penicillin/streptomycin (P/S; Thermo Fisher Scientific, Illkirch-Graffenstaden, France) in a humidified atmosphere at 37 °C with 5% CO_2_.

### 2.2. Virus

ZIKV was rescued by transfection of 200 ng the infectious clone in 50% confluent HEK-293T cells (seeded the previous day in a six-well plate) using TransIT-LT1 transfection reagent (Mirus Bio, Strasbourg, France). Four days after transfection, the supernatant was clarified by centrifugation, virus titer determined by plaque assay and frozen at −80 °C. The virus stock used in this study was generated by infection of Vero cells with rescued ZIKV, at an MOI of 0.001 PFU/cell. Five days post infection, the supernatant was clarified by centrifugation, virus titer determined by plaque assay and frozen at −80 °C prior to use in the growth curves.

### 2.3. Plaque Assay

Viral titration was performed on Vero cells plated 1 day prior to infection on 24 well plates. Ten fold dilutions were performed in DMEM alone and transferred onto Vero cells for 1 h to allow infection before adding DMEM with 2% FCS, 1% P/S and 0.8% agarose. Plaque assays were fixed with 4% formalin (Sigma-Aldrich, Saint-Quentin-Fallavier, France) 4 days p.i. (ZIKV) and plaques were manually counted.

### 2.4. Decay Curves

Decay curves were carried out in the absence of cells. Briefly, viral plaque-forming units equivalent to low or high MOI infections described below (growth curves) were placed in 12-well plates in triplicate, and incubated at 37 °C. Virus was diluted in 1 mL cell culture media supplemented with 2% FCS. At each time point (0 h, 4 h, 6 h, 8 h, 24 h, 48 h, 72 h, and 96 h), 60 μL and 5 μL were separately aliquoted and frozen for further titration and RT-qPCR. 65 μL of fresh media was added to replace the taken volume.

### 2.5. Growth Curves

Cells were plated in 12 well plates at 80–90% confluence one day before infection. At day 0, virus was diluted in 300 μL PBS to obtain a multiplicity of infection (MOI) of 1 PFU per cell (high MOI) or 0.01 PFU per cell (low MOI). After 1 h, the viral solution was removed, cells were washed three times with PBS and new media (1 mL) supplemented with 2% FCS was added. At each time point 0 h, 4 h, 6 h, 8 h, 24 h, 48 h, 72 h, and 96 h for ZIKV infection 60 μL and 5 μL were separately aliquoted and frozen for further titration and RT-qPCR. 65 μL of fresh media was added on top of cells to replace the taken volume. Each growth curve was done in triplicates.

### 2.6. RT-qPCR

As described in [43], cell supernatants were heated 5 min at 60 °C for viral inactivation. Quantitative RT-PCR (RT-qPCR) was then performed with TaqMan RNA-to-Ct One-step RT-PCR kit (Applied Biosystems, Thermo Fisher Scientific, Illkirch-Graffenstaden, France) using the following cycling conditions: 20 min at 50 °C, 10 min at 95 °C, 40 cycles of 95 °C for 15 s, followed by 60 °C for 1 min). The primer and probe sets used for each virus are shown in Table 3. RNA copy number was derived from a standard curve generated using reactions containing 10-fold dilutions of known amounts of in vitro generated RNA transcripts Each reaction contained a scale of diluted IVT to calculate RNA copy number. The ZIKV primers bind to and amplify a 77 nucleotide region in the 5${}^{\prime }$ end of the ZIKV genome (position 1192-1268). The primers and the problem used in RT qPCR are given in Table 1.

### 2.7. Degradation of Encapsulated Genome and Infectious Virus

**Figure 1:**
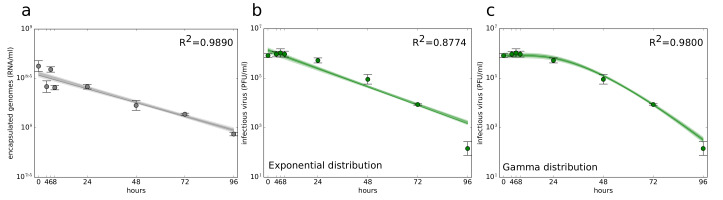
Time course decay of Zika virus (ZIKV) in the physical conditions of the in vitro kinetic experiments. Decay of (**a**) encapsulated genomes and (**b**,**c**) infectious virus described by (**b**) exponential distribution decay model (2) with $n_{\mathrm{pfu}}=1$ and (**c**) gamma distribution decay model (2) with $n_{\mathrm{pfu}}=8$. Data are shown as the mean ± standard deviation. The best-fits are displayed as solid green lines. The light shading around the best-fits corresponds to the model kinetics associated with MCMC-accepted parameters. The dark shading represents 95% credible region.

Stability of encapsulated genomes (Figure 1a) was quantified following the assumption that virus particles degrade in an exponential manner over time, which was mathematically expressed as

(1) $$\begin{array}{cc} \frac{dV^{\mathrm{rna}}}{dt} & =-\frac{1}{\tau_{\mathrm{rna}}}\;V^{\mathrm{rna}}, \end{array}$$

where $\tau_{\mathrm{rna}}$ (measured in (h)) is an average time for a viral genome to lose stability.

Experimental data showed that ZIKV does not lose infectivity in the first several hours (Figure 1b,c). This suggests that the standard assumption of exponentially distributed viral decay times is not appropriate to describe the loss of ZIKV infectivity. Therefore, we introduced ‘aging’ of the infectious virus and separated infectious virus lifespan ($\tau_{\mathrm{pfu}}$) into ($n_{\mathrm{pfu}}$) stages, each of which last for an exponentially-distributed time of equal average length ($\tau_{\mathrm{pfu}}/n_{\mathrm{pfu}}$) (measured in hours (h)). For $n_{\mathrm{pfu}}=1$, the infectious virus lifespan was exponentially distributed, for $n_{\mathrm{pfu}}>1$ the infectious virus lifespan followed gamma distribution. Only infectious virus in the last stage $V_{n_{\mathrm{pfu}}}^{\mathrm{pfu}}$ was allowed to lose infectivity completely. A similar approach was used to describe the duration of eclipse and virus-producing phases of cells infected with SHIV [20,21] or influenza A virus [32]. Equations describing the loss of virus infectivity are as follows

(2) $$\begin{array}{cc} \frac{dV_{1}^{\mathrm{pfu}}}{dt} & =-\frac{n_{\mathrm{pfu}}}{\tau_{\mathrm{pfu}}}\;V_{1}^{\mathrm{pfu}},\\ \frac{dV_{k=2,\ldots ,n_{\mathrm{pfu}}}^{\mathrm{pfu}}}{dt} & =\frac{n_{\mathrm{pfu}}}{\tau_{\mathrm{pfu}}}\;\left(V_{k-1}^{\mathrm{pfu}}-V_{k}^{\mathrm{pfu}}\right). \end{array}$$

### 2.8. Mathematical Model of ZIKV In Vitro Kinetics

Time course ZIKV kinetics were numerically simulated using a viral dynamics mathematical model that was develped and used to describe in vitro and in vivo infection of influenza A virus and its variants [32,36], SHIV [20,21], or respiratory syncytial virus [10] as well as the interactions between a fully functional virus and its defective interfering particles [44,45]. In this model, susceptible target cells (*T*) become infected by infectious virus at any infectious stage ($V_{k=1,\ldots ,n_{\mathrm{pfu}}}^{\mathrm{pfu}}$), measured in plaque forming units per millilitre (PFU/mL) at the infection rate (β) (measured in mL × (PFU × h)${}^{-1}$). Upon successful infection, target cells enter an eclipse phase (the time between virus entry into the cell to the beginning of viral release out of the cell) which is separated into ($n_{E}$) stages. Eclipse cells ($E_{i=1,\ldots ,n_{E}}$) remain in each stage $i=1,\ldots ,n_{E}$ for an exponentially-distributed time of equal average length ($\tau_{E}/n_{E}$) (measured in hours (h)). Only eclipse cells in the last compartment ($E_{n_{E}}$) are allowed to transition into the infectious state and begin producing viral genomes. Infectious phase (the amount of time between the beginning and end of viral release out of a cell) is separated into ($n_{I}$) stages, and infectious cells ($I_{j=1,\ldots ,n_{I}}$) spend an exponentially-distributed time of equal average length ($\tau_{I}/n_{I}$) (measured in h) in each stage before infectious cells in the last stage ($n_{I}$) are removed from the system. Infectious cells in all stages can produce infectious virus in the first infectious stage ($V_{1}^{\mathrm{pfu}}$) at the rate ($p_{\mathrm{pfu}}$) (measured in PFU × (cell × mL × h)${}^{-1}$) and encapsulated genomes ($V^{\mathrm{rna}}$) (measured in RNA/mL) at the rate ($p_{\mathrm{rna}}$) (measured in RNA × (cell × mL × h)${}^{-1}$). The lifespan of infectious virus is separated into ($n_{\mathrm{pfu}}$) stages, each of which last for an exponentially-distributed time of equal average length ($\tau_{\mathrm{pfu}}/n_{\mathrm{pfu}}$) (measured in h). Encapsulated genomes remain stable for an average time ($\tau_{\mathrm{rna}}$) (measured in h). We also assume that infectious virus ($V_{k}^{\mathrm{pfu}}$) in any stages $k=1,\ldots ,n_{\mathrm{pfu}}$ infects susceptible cells at the same rate. We do not consider coinfection or superinfection of already infected cells; once a cell is infected, no more infectious virus can enter. The full model is given as

(3) $$\begin{array}{cc} \frac{dT}{dt} & =-\beta \;T\;\sum_{k=1}^{n_{\mathrm{pfu}}}V_{k}^{\mathrm{pfu}},\\ \frac{dE_{1}}{dt} & =\beta \;T\;\sum_{k=1}^{n_{\mathrm{pfu}}}V_{k}^{\mathrm{pfu}}-\frac{n_{E}}{\tau_{E}}\;E_{1},\\ \frac{dE_{i=2\ldots n_{E}}}{dt} & =\frac{n_{E}}{\tau_{E}}\;\left(E_{i-1}-E_{i}\right),\\ \frac{dI_{1}}{dt} & =\frac{n_{E}}{\tau_{E}}\;E_{n_{E}}-\frac{n_{I}}{\tau_{I}}I_{1},\\ \frac{dI_{j=2\ldots n_{I}}}{dt} & =\frac{n_{I}}{\tau_{I}}\;\left(I_{j-1}-I_{j}\right),\\ \frac{dV_{1}^{\mathrm{pfu}}}{dt} & =p_{\mathrm{pfu}}\;\sum_{j=1}^{n_{I}}I_{j}-\frac{n_{\mathrm{pfu}}}{\tau_{\mathrm{pfu}}}\;V_{1}^{\mathrm{pfu}},\\ \frac{dV_{k=2\ldots n_{\mathrm{pfu}}}^{\mathrm{pfu}}}{dt} & =\frac{n_{\mathrm{pfu}}}{\tau_{\mathrm{pfu}}}\;\left(V_{k-1}^{\mathrm{pfu}}-V_{k}^{\mathrm{pfu}}\right),\\ \frac{dV^{\mathrm{rna}}}{dt} & =p_{\mathrm{rna}}\sum_{j=1}^{n_{I}}I_{j}-\frac{1}{\tau_{\mathrm{rna}}}\;V^{\mathrm{rna}}. \end{array}$$

The experiments to obtain viral load time course datasets began with overlaying the virus supernatant on susceptible cells followed by a one-hour cultivation to allow cell infection. The supernatant was then removed and cells were thoroughly washed off the remaining virus. However, despite the repeated washing, we still detected residual infectious virus in the supernatant. We utilized these data in modelling ZIKV in vitro kinetics by assuming that such residual virus ($V_{k=1\ldots n_{\mathrm{pfu}}}^{{\mathrm{pfu}}_{\mathrm{res}}}$) did not engage in the virus-cell interactions and was only allowed to decay according to the following dynamics

(4) $$\begin{array}{cc} \frac{dV_{1}^{{\mathrm{pfu}}_{\mathrm{res}}}}{dt} & =-\frac{n_{\mathrm{pfu}}}{\tau_{\mathrm{pfu}}}\;V_{1}^{{\mathrm{pfu}}_{\mathrm{res}}},\\ \frac{dV_{k=2\ldots n_{\mathrm{pfu}}}^{{\mathrm{pfu}}_{\mathrm{res}}}}{dt} & =\frac{n_{\mathrm{pfu}}}{\tau_{\mathrm{pfu}}}\;\left(V_{k-1}^{{\mathrm{pfu}}_{\mathrm{res}}}-V_{k}^{{\mathrm{pfu}}_{\mathrm{res}}}\right). \end{array}$$

To define the initial conditions for the system (3) and (4), we used the results of generalized target theory [46]. Given the multiplicity of infection (MOI, the ratio of infectious virus in the inoculum to the total number of susceptible cells), the proportion of susceptible cells that would receive any number of infectious viruses (*N*) follows Poisson distribution [47]:

(5) $$\mathrm{Proportion}\;\mathrm{of}\;\mathrm{cells}\;\mathrm{receiving}\;N\;\mathrm{infectious}\;\mathrm{viruses}=\frac{{\mathrm{MOI}}^{N}\;\exp\left(-\mathrm{MOI}\right)}{N!}.$$

We further allow only eclipse cells in their first stage, $E_{1}$, to have received the virus. Therefore, the proportion of cells in the first phase, $E_{1}$, which received one or more infectious viruses is equivalent to the total proportion of cells excluding those which did not receive any infectious virus

(6) $$\mathrm{Proportion}\;\mathrm{of}\;E_{1}\;\mathrm{cells}=1-\frac{{\mathrm{MOI}}^{0}\;\exp\left(-\mathrm{MOI}\right)}{0!}=1-\exp\left(-\mathrm{MOI}\right).$$

Thus, the initial conditions are $T\left(t=0\right)=T_{0}\times \exp\left(-\mathrm{MOI}\right)$, $E_{1}\left(0\right)=T_{0}\times \left(1-\exp\left(-\mathrm{MOI}\right)\right)$, $E_{2,\ldots ,n_{E}}\left(0\right)=0$, $I_{1,\ldots ,n_{I}}\left(0\right)=0$, $V_{1,\ldots ,n_{\mathrm{pfu}}}^{\mathrm{pfu}}\left(0\right)=0$, $V_{2,\ldots ,n_{\mathrm{pfu}}}^{{\mathrm{pfu}}_{\mathrm{res}}}\left(0\right)=0$ and $V_{\mathrm{rna}}\left(0\right)=0$, where $T_{0}=2\times 10^{5}$ susceptible cells seeded in each well. The parameter $V_{1}^{{\mathrm{pfu}}_{\mathrm{res}}}\left(0\right)$ is a free parameter to be estimated.

### 2.9. Selection of Data Points for Parameter Estimation

Viral load measurements, both infectious virus (measured in PFU/mL) and encapsulated genomes (measured in RNA/mL) which fell below the limit of detection for a given quantification method were excluded from parameter estimation routine. The limit of detection of the infectious virus was 10${}^{2}$ PFU/mL. The limit of detection of encapsulated genomes was set to 10${}^{6}$ RNA/mL which was equivalent to RT-qPCR Ct value equal or greater than 30.

## 3. Results

### 3.1. Quantification of ZIKV Stability Determinants

The capacity of the virus to invade the host cell and exploit its resources to replicate and produce infectious progeny is time-limited and may have significant impacts on the overall viral dynamics. To precisely calculate the average time for infectious virus to lose infectivity $\tau_{\mathrm{pfu}}$ and for encapsulated genomes to lose stability $\tau_{\mathrm{rna}}$, we incubated ZIKV stock at 37 °C for up to 96 h in cell culture media. At 0 h, 4 h, 6 h, 8 h, 24 h, 48 h, 72 h, and 96 h, RNA was extracted for quantification by reverse transcription quantitative polymerase chain reaction (qRT-PCR) to determine total encapsulated genome concentration and infectious virus remaining in the media was quantified by plaque assay.

The ZIKV encapsulated genomes degraded slowly over time. This process was well described by the model (1) assuming an exponentially distributed degradation time ($\tau_{\mathrm{rna}}$). By fitting Equation (1) to encapsulated genome concentration time course data, we determined that ZIKV genome degradation time ($\tau_{\mathrm{rna}}$) was 74.86 h (Figure 1a). The initial condition for encapsulated genome concentration ($V_{0}^{\mathrm{rna}}$) was left as a free parameter. We extracted the parameter distributions and 95% credible regions (CrRs) for the parameters using the module *emcee* [48], an implementation of the Markov chain Monte Carlo (MCMC) method [49] in Python (Python Software Foundation, Python Language Reference, version 2.7, available at https://www.python.org/). This approach of using the MCMC and the Python module *emcee* to generate the parameter posterior distributions and 95% CrRs for parameters of their in vitro mathematical models was introduced by Beauchemin and colleagues [10,21,36]. The values of $\tau_{\mathrm{rna}}$ were found to be within [70.31, 76.43] h. The corresponding parameter posterior distributions and 95% credible regions are in Figure S1 in Supplementary Materials File S1. The best-fit parameter values and their corresponding 95% CrIs are given in Table 2. Details of the fitting and MCMC procedures are in Supplementary Materials File S1.

**Figure 2:**
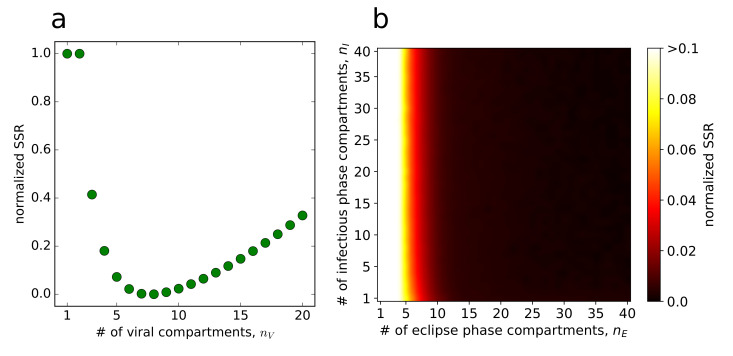
Selecting the number of compartments $n_{\mathrm{pfu}}$, $n_{E}$ and $n_{I}$. For each $n_{\mathrm{pfu}}$ and a pair ($n_{E},n_{I}$), we calculated the relative value of the objective function obtained from fitting as a fraction (SSR − SSR${}_{\min}$)/(SSR${}_{\max}$− SSR${}_{\min}$), where SSR (sum of squared residuals) is the best-fit value of the objective function for a given $n_{\mathrm{pfu}}$ or a pair ($n_{E},n_{I}$) and SSR${}_{\max}$ and SSR${}_{\min}$ are the lowest and highest values out of all objective function values. The normalized values range between 0 (best fits) and 1 (worst fits). (**a**) To determine $n_{\mathrm{pfu}}$, and thus the number of compartments in the decay model (2), Formula (S4) in Supplementary Materials File S1 was used. Here, the maximum and minimum values of the objective function (S4) over all $n_{\mathrm{pfu}}$ were SSR${}_{\max}=124.29$ and SSR${}_{\min}=28.33$. (**b**) To determine $n_{E}$ and $n_{I}$, and thus the number of eclipse and infectious compartments in the model (3), Equation (S7) in Supplementary Materials File S1 was used. Here, the maximum and minimum values of the objective function (S7) over all pairs ($n_{E},n_{I}$) were SSR${}_{\max}=589.9$ and SSR${}_{\min}=39.28$.

Infectivity of ZIKV remained unchanged for the first 8 hours and began to notably decrease after 24 h (Figure 1b,c). This suggests that infectious ZIKV does not decay in an exponential manner. Data indicated that there is a delay in infectious ZIKV decay. We modelled this delay by introducing ($n_{\mathrm{pfu}}$) compartments so that infectious virus spends a time of ($\tau_{\mathrm{pfu}}/n_{\mathrm{pfu}}$) in each compartment $i\;=\;1,\ldots ,n_{\mathrm{pfu}}$ before transitioning into the compartment i+1. Since the amount of time spent in each stage is exponentially distributed, the time during which virus remains infectious is described by the sum of ($n_{\mathrm{pfu}}$) exponential distributions, and in our case, by gamma distribution [50]. The model is given by Equation (2) (Materials and Methods), in which $n_{\mathrm{pfu}}=1$ gives an exponential decay of infectious ZIKV, whereas $n_{\mathrm{pfu}}>1$ gives a gamma-distributed decay of infectious ZIKV. To quantify the decay time ($\tau_{\mathrm{pfu}}$), we varied ($n_{\mathrm{pfu}}$) over a range of integer values from 1 to 20 and fit the model (2) to the time course infectious virus degradation measurements (details on the fitting scheme are in Supplementary Materials File S1). We set the initial virus concentration in the first stage, ($V_{1}^{\mathrm{pfu}}\left(0\right)$), as a free parameter and in the remaining stages ($V_{k=2,\ldots ,n_{\mathrm{pfu}}}^{\mathrm{pfu}}\left(0\right)$) to zero. We discriminated between the individual fits based on the values of their associated objective functions and determined that the number of compartments associated with the overall best-fit of the model (2) was $n_{\mathrm{pfu}}=8$. This is graphically captured in Figure 2a, in which the scaled value of the objective function is plotted against the number of viral compartments $n_{\mathrm{pfu}}$ and has a minimum at $n_{\mathrm{pfu}}=8$. Interestingly, for $n_{\mathrm{pfu}}=1$, we obtained the worst fit of the model (2) in terms of objective function. This was also corroborated visually by comparing the infectious ZIKV decay dynamics yielded by the model (2) for $n_{\mathrm{pfu}}=1$ and $n_{\mathrm{pfu}}=8$ (Figure 1b,c, respectively), as well as the associated coefficients of determination $R^{2}=0.8774$ and $R^{2}=0.9800$, respectively. The gamma distribution decay model performed significantly better than the exponential one also in terms of the MCMC associated *p*-value (*p*< 0.005). We determined the degradation time of infectious ZIKV $\tau_{\mathrm{pfu}}$ to be 39.55 h and 95% CrIs [38.93, 40.22] h (Table 3). The posterior distributions of decay parameters are depicted in Figure S1 in Supplementary Materials File S1. We note that Kakizoe and colleagues [20] propose other distributions that describe durations of eclipse and virus-producing phases of infected cells and could describe ZIKV decay. We discuss a model, in which the decay time is Weibull distributed in Supplementary Materials File S1.

### 3.2. Experimental Time Course Kinetics of ZIKV Infection In Vitro

**Figure 3:**
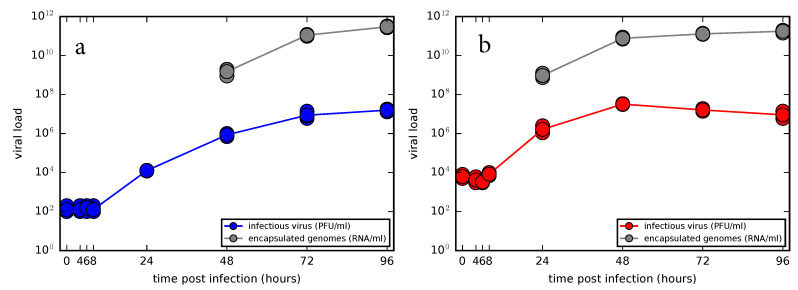
Time course kinetics of infectious virus and encapsulated genome concentrations for two distinct initial viral concentrations. (**a**) Multiplicity of infection (MOI) 0.01 and (**b**) 1 infectious units per cell.

To study the time course of ZIKV infection in vitro, characterize the shape of viral load kinetic curves and evaluate viral kinetic parameters, we conducted two infection experiments in Vero cells using two distinct initial viral concentrations with the multiplicity of infection (MOI) 0.01 and 1 infectious units per cell, hereafter referred to as low and high MOI infections, respectively (Figure 3). Since Vero cells are an interferon-deficient mammalian cell line incapable of secreting type I interferon in response to viral infection, we did not assume any immune response of that type in our model and focused only on the interactions between the virus and cells.

The viral load kinetics appeared to proceed in a similar manner for both low and high MOI infections. In the first four measured time points, 0 h, 4 h, 6 h, and 8 h the virus titers remained unchanged which suggests that no production of the new infectious virus occurred during that time period. The lack of decay dynamics in the first 8 h also reflects our observation from the decay experiment, that is, infectious ZIKV does not lose infectivity in the first 8 h, as depicted in Figure 1b,c. At 24 h, viral titers increased approximately a hundred-fold as the virus completed its replication cycle in the cells infected by the inoculum. In the case of low MOI infection, viral titers continued to grow up to 96 h, though the accumulation slowed down towards the end of the experiment. In the case of high MOI infection, viral titers peaked at 48 h and began to slowly decline as cells stopped producing new virions and the remaining infectious extracellular virus began to degrade. The encapsulated genome concentration in the medium as quantified by qRT-PCR appeared to mirror the time course kinetics of infectious virus, though for the high MOI infection the accumulation of genomes slowed down at 48 h, coinciding with the peak of infectious virus.

### 3.3. Quantification of ZIKV Life-Cycle Determinants

**Figure 4:**
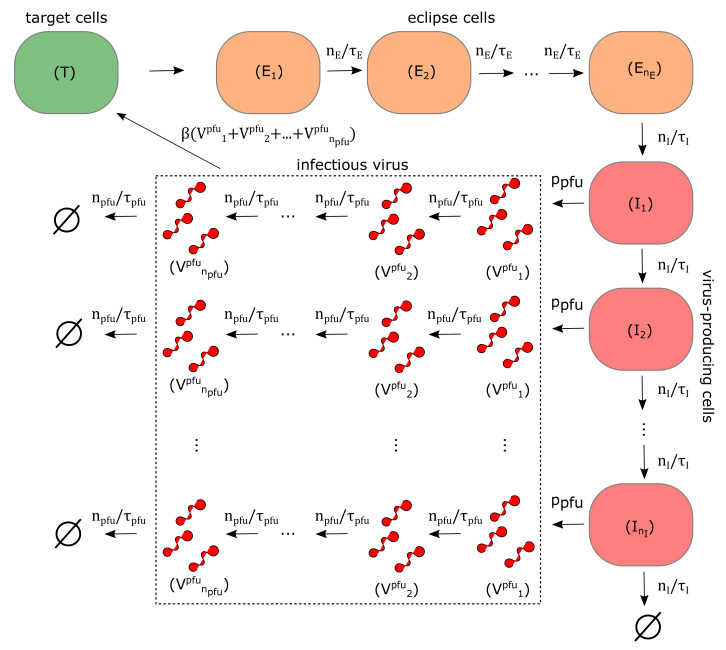
Graphical representation of the model (3) described in Materials and Methods. Susceptible target cells (green) get infected by infectious virus at any stage of its infectious life (dotted rectangle). After entering an eclipse phase, eclipse cells (orange) remain in the eclipse phase for an average time ($\tau_{E}$), after which they transition into the virus-producing phase and begin releasing virus. Virus-producing cells (red) remain in virus-producing state for an average time ($\tau_{I}$) and produce infectious virus at the rate ($p_{\mathrm{pfu}}$) and total encapsulated genomes at the rate (($p_{\mathrm{rna}}$) the latter is not displayed). Infectious virus remains infectious for an average time ($\tau_{\mathrm{pfu}}$). Encapsulated viral genomes remain stable for an average time (($\tau_{\mathrm{rna}}$), not displayed).

We numerically quantified the main determinants of ZIKV in vitro kinetics, in the model (3) (graphically captured in Figure 4) represented by the rate of infection via infectious virus (β), the length of eclipse phase ($\tau_{E}$), the length of virus-producing phase ($\tau_{I}$), the rate of infectious virus production ($p_{\mathrm{pfu}}$), the rate of total encapsulated genome production ($p_{\mathrm{rna}}$) and two additional free parameters describing the initial concentration of infectious virus remaining in the well after washing ($V_{1}^{{\mathrm{pfu}}_{\mathrm{res}}}\left(0\right)$), which we will denote as ($V_{l}^{\mathrm{pfu}}\left(0\right)$) for low-MOI infection and ($V_{h}^{\mathrm{pfu}}\left(0\right)$) for high-MOI infection. The residual infectious virus was not considered to engage in the cell infection and was only allowed to decay. This dynamics were described by Equation (4).

In the Equation (3), cells in the eclipse and virus-producing phases move through multiple stages, ($n_{E}$) and ($n_{I}$), respectively, before they transition into the virus-producing phase and are removed from the virus-cell interactions, respectively. This type of multi-staged model, in which only the cells in the last stage of virus-producing phase are allowed to disintegrate, was introduced by Beauchemin and colleagues [10,32,36]. In their work, they decided the number of compartments for the eclipse and virus-producing phases by evaluating the sensitivity of the model to changes ($n_{E}$) and ($n_{I}$) and chose those values of $n_{E}$ and $n_{I}$ for which the model yields an overall best fit [10] or, alternatively, the highest possible values $n_{E}$ and $n_{I}$ were selected if the model proved insensitive to changes in these parameters [32]. Similarly to these studies, we decided the number of compartments for eclipse and virus-producing phases, for each pair $\left(n_{E},n_{I}\right)$, both ranging from 1 to 40, by simultaneously fitting the model (3) and (4) to low and high MOI viral load data fifty times, each time initiating the procedure from a different set of values for β, $\tau_{E}$, $\tau_{I}$, $p_{\mathrm{pfu}}$, $p_{\mathrm{rna}}$, $V_{l}^{\mathrm{pfu}}\left(0\right)$ and $V_{h}^{\mathrm{pfu}}\left(0\right)$. We then selected the parameter set that was associated with the overall lowest value of the objective function (given by the Formula (S7) in Supplementary Materials File S1) as the best-fit parameter set for a given pair $\left(n_{E},n_{I}\right)$. Details of the fitting scheme are given Supplementary Materials File S1.

Figure 2b depicts the scaled values of the objective function for each pair ($n_{E},n_{I}$) as a heatmap. Considerably better fits of the model to the data were achieved for higher values of ($n_{E}$) compared to lower values of ($n_{E}$) (horizontal axis in Figure 2b), though for $n_{E}\gg 1$, the model was insensitive to changes in ($n_{E}$). This result aligns with the results obtained for other enveloped viruses, such as influenza A virus, SHIV, and studies in which delays in the eclipse to virus-producing transitions were investigated [20,34,50,51]. The model was insensitive to changes in ($n_{I}$) (vertical direction in Figure 2b). Due to insensitivity of the model to the precise values of $n_{E}$ and $n_{I}$, we fixed $n_{E}$ and $n_{I}$ to their maximal allowed values $n_{E}=n_{I}=40$, as was done in [36], to avoid a biologically implausible scenario, in which cells would be allowed to initiate and stop releasing viral particles instantly after they become infected and enter the virus-producing phase, respectively, which would be possible if the exponentially distributed duration of both phases were allowed [32].

**Figure 5:**
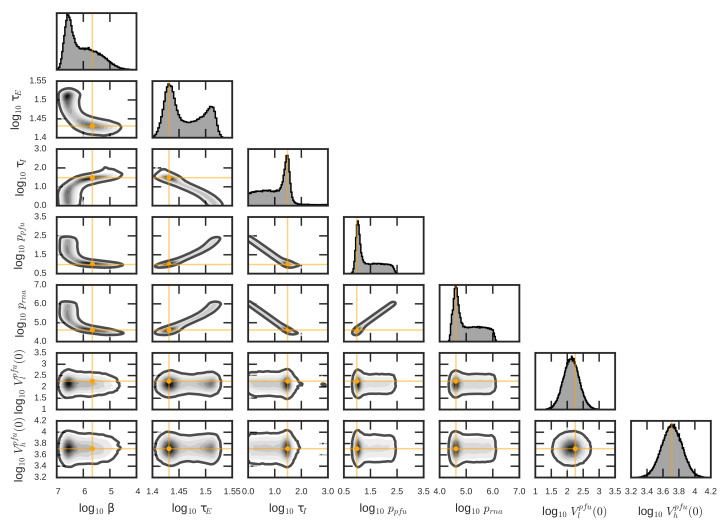
Parameter posterior distributions and pair-wise posterior plots obtained from MCMC run of the model (3) and (4). The orange targets indicate the best-fit parameter values given in Table 4. The solid dark lines enclose the 95% credible regions.

The best-fit parameters values of the model (3) for the selected pair $\left(n_{E},n_{I}\right)=\left(40,40\right)$ are given in Table 4. We again used the MCMC to evaluate 95% credible regions and determine posterior distributions for all model parameters (given also in Table 4). These are captured in Figure 5. The MCMC revealed correlations between the model parameters. The length of the virus-producing phase ($\tau_{I}$), the posterior distribution of which had a peak at around the best-fit value $\tau_{I}=30.4$ h and a flat left tail capturing a shorter virus-producing phase, was strongly correlated with the infectious virus and total encapsulated genome production rates, ($p_{\mathrm{pfu}}$) and ($p_{\mathrm{rna}}$), respectively. These rates showed peaks at around the best-fit values 9.65 PFU/(cell × mL × h) and $4.11\times 10^{4}$ RNA/(cell × mL ×h), respectively and exhibited flat right tails, representing even faster production rates. Here, the experimental data could not inform the model to distinguish between having more virus produced during a short virus-producing phase (high ($p_{\mathrm{pfu}}$) and ($p_{\mathrm{rna}}$), low ($\tau_{I}$)) and having less virus produced during a longer virus-producing phase (low ($p_{\mathrm{pfu}}$) and ($p_{\mathrm{rna}}$), high ($\tau_{I}$)), resulting in a negative correlation between production rates and the length of virus-producing phase). The length of the eclipse phase ($\tau_{E}$), the best-fit value of which was 27 h, was found to be negatively correlated with the length of the virus-producing phase ($\tau_{I}$) as longer eclipse phase is be balanced out by a shorter virus-producing phase (and higher viral production rates) to reproduce the data. Similar reasoning can be applied to explain correlations for the remaining viral parameters.

**Figure 6:**
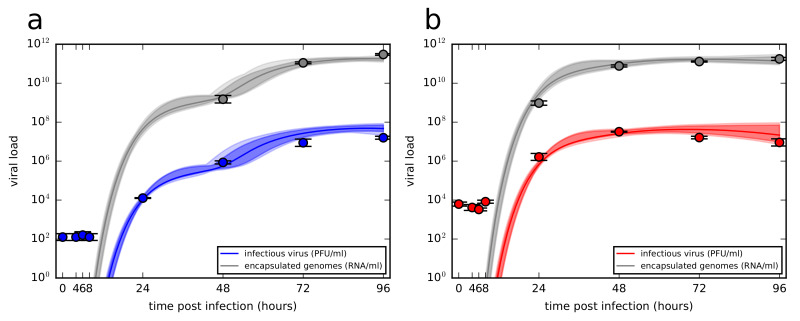
Time course of ZIKV infectious virus load and encapsulated genomes for (**a**) low (blue) and (**b**) high (red) MOI viral load data. Data are shown as the mean ± standard deviation. The best-fits are displayed as solid green lines. The light shading around the best-fits corresponds to the model kinetics associated with MCMC-accepted parameters. The dark shading represents 95% credible region.

The time course infection kinetics of newly produced ZIKV yielded by the model (3) and (4) is depicted in Figure 6. In the case of low MOI infection (Figure 6a), the model allowed for multi-step virus kinetics when the first round of virus released from the cells initially infected by the inoculum was followed by the second round of viral release out of the cells infected by a newly produced virus, here represented by two subsequent influxes in viral load. In the case of high MOI infection (Figure 6b), single-step virus kinetics was reproduced by the model depicting a sharp increase in virus load within 48 h accumulated during the synchronized infection of the majority of initially infected cells. We note that continuous sampling of viral supernatant for quantification at each measured time had only negligible effects on the model output (Figure S3, details are in Supplementary Materials File S1) and thus was omitted in the simulations. Overall, the presented model was able to deliver a realistic description of ZIKV in vitro infection experimental data.

## 4. Discussion

The combined approach of experimental and mathematical modelling allowed us to characterize the ZIKV infection kinetics in vitro, the aspects of which remained unexplored. Experimental data we generated showed that the inactivation of infectious ZIKV particles does not proceed in an exponential manner. We introduced a mathematical model of infectious virus degradation dictated by gamma distribution which was able to explain the lack of decay dynamics in the first 24 h followed by a decline onwards. We estimated the decay time of infectious ZIKV (MR766 strain) to be almost 40 h, which was longer compared to the previously reported 7.5 h (when back-calculated from the half-life of 5.2 h, assuming an exponential decay model) [13]. The observed discrepancies in the ZIKV infectivity could be explained by different techniques used to quantify the infectious virus. We measured the amount of infectious virus left in the stock in the cell line that we used to perform plaque assay, that is, Vero cells, whereas in [13] flow cytometry was performed on ZIKV-infected Raji-DC-SIGN-R cells. This allows to measure the ability of a virus to infect a cell for one cycle, but not necessarily to remain infectious and infect neighbouring cells. This is, however, essential when measuring infectious virus that can re-infect new cells in the context of a growth curve.

We quantified the in vitro ZIKV infection kinetics and revealed correlations between the viral parameters. The low values for the length of the virus-producing phase of infected cells ($\tau_{I}$) (short virus-producing phase) were compensated by increased viral production rates, resulting in negative correlations between those parameters. We hypothesize that the inability of the model to fully identify the length of the virus-producing phase ($\tau_{I}$) could be due to an insufficient number of data points collected during the late phase of infection. Our experiment was terminated at 96 h post-inoculation when the viral load is still on the increase in the case of low MOI infection and peaking and slowly declining in the case of high MOI infection. To be able to fully capture the post-peak decline, the timespan of the experiment would need to double, given the very slow accumulation of infectious ZIKV. Another possibility to overcome this problem would have to include collecting timely measurements of susceptible, infected and dead cells. Therefore, identifying the times and frequencies with which viral load measurements need to be collected is crucial, as these are usually more accessible to mathematical modellers.

Previous experimental work showed late-onset apoptosis 48 h after infection of human lung epithelial A549 cells with ZIKV isolate PF13 (previously described in [52]), which occurred at a slower rate compared to the course of viral production. Delayed apoptosis of A549 and Vero cells were also reported for the viral molecular clone of the epidemic strain from Asian lineage, BeH819015 isolated in Brazil in 2015, which occurred after the maximum production of viral progeny [53]. These experimental observations are in good agreement with our experimental and modelling results as the model predicts the best-fit value for the length of the virus-producing phase ($\tau_{I}$) to be 30.4 h with the peak of viral production to be between 24 and 48 h. This could suggest that the left tail of the posterior distribution of ($\tau_{I}$) does not reflect realistic durations of the virus-producing phase. Although not all cells at the end of their virus-producing phase will undergo apoptosis, considering such approximation in the mathematical model will help to predict and quantify the timing of ZIKV-induced cellular death.

The viral dynamics model (3) assumes that all virus-producing cells in each stage produce infectious virus and encapsulated viral genomes at the same (maximum) rates ($p_{\mathrm{pfu}}$) and ($p_{\mathrm{rna}}$), respectively. However, not all cells in the virus-producing stage will begin releasing virus at the same rate. This might be influenced either by the multiplicity of cellular infection or phenotypic heterogeneity in the cell population. The effects of multiple infection of cells with different copies of the same virus on the virus kinetics was described in [54], while the effects of increased viral replication in multiple-infected cells was studied in [55]. One can generalize the terms for release of virus in the model (3) by introducing the terms $\sum_{j=1}^{n_{I}}p_{\mathrm{pfu},\;j}\;I_{j}$ and $\sum_{j=1}^{n_{I}}p_{\mathrm{rna},\;j}\;I_{j}$, in which cells in different stages $j=1,\ldots ,n_{I}$ exhibit different production rates ($p_{\mathrm{pfu},\;j}$) and ($p_{\mathrm{rna},\;j}$). A delay between productive infection of cells and virus release can be modelled by setting $p_{\mathrm{pfu},\;j}=0$ and $p_{\mathrm{rna},\;j}=0$ for the first *m* stages [50]. The age of productively infected cells might influence the virus production rates, as suggested by [56], with the youngest and oldest cells experiencing low to no production of virions. In our model, this could be approximated by setting the production rates to zero for the first *m* and for the last $n_{I}-M$ stages, where m<M. These conditions could implicitly account for the limitations in cellular resource availability during the viral replication cycle, particularly towards the end of the virus-producing phase of infected cells.

Predictions from our mathematical model are limited to the studied ZIKV strain (African lineage MR-766) and the cell line used to study viral replication kinetics (African green monkey kidney Vero cells). The replication kinetics of different ZIKV isolates were experimentally investigated to characterize lineage-specific phenotypes. Asian and African ZIKV isolates were reported to display minimal lineage-specific differences in their growth curves in Vero cell line [57,58]. In contrast, the African strain ArD 41525 was described to consistently exhibit faster replication kinetics not only in the Vero cell line but also in HEK-293 (human), DEF (avian), and RK-13 (rabbit) cells lines [59]. Therefore, it is not straightforward to generalize the results from our mathematical model as different strains can exhibit distant growth kinetics or the same strain may perform differently in various cell lines. For example, ZIKV was shown to accumulate slower in mosquito cells compared to mammalian cells [57]. This was attributed to different physical conditions that are required for the cultivation of mammalian and mosquito cells, as the latter are incubated at lower temperatures (usually 28 °C) than the former (usually 37 °C). In addition, inactivation of infectious ZIKV could be affected by different temperatures, too, as reported in [12]. Thus, strain-specific and host cell-specific modelling studies would be desirable to identify and quantify these specificities, which would strengthen our understanding of the main drivers of ZIKV-host cell iterations.

In this study, we described the in vitro kinetics of ZIKV, a member of the Flavivirus genus, which remained largely unexplored, by applying the synergistic combination of mathematical modelling and experimental data. Our study thus represents a step to quantitatively elucidate the in vitro dynamics of ZIKV infection in a manner that is inaccessible through conventional experimental approaches.

## Figures and Tables

**Table 1:** The primer and probe sets used in reverse transcription quantitative polymerase chain reaction (RT-qPCR) to quantify ZIKV encapsulated genomes.

Forward primer (5${}^{\prime }$ to 3${}^{\prime }$)	TCGTTGCCCAACACAAG
Reverse primer (5${}^{\prime }$ to 3${}^{\prime }$)	CCACTAATGTTCTTTTGCAGACAT
Probe (5${}^{\prime }$ [6-FAM] to 3${}^{\prime }$)	GCCTACCTTGACAAGCAATCAGACACTCA

**Table 2:** Parameter values obtained from fitting Equation (1) to encapsulated genome data. 95% CrRs were constructed from Markov chain Monte Carlo (MCMC) fits of the model (1) to encapsulated genome data.

Parameter	Description	Units	Value	95% CrR
$\tau_{\mathrm{rna}}$	decay time of encapsulated genomes	h	74.86	[70.31, 76.43]
$V_{0}^{\mathrm{rna}}$	initial concentration of encapsulated genomes	$\times 10^{8}$ RNA/mL	3.42	[3.38, 3.65]

**Table 3:** Parameter values obtained from fitting Equations (2) to viral titer data. The 95% credible regions (CrRs) were constructed from MCMC fits of the model (2) to data.

Parameter	Description	Units	Value		95% CrR	
$n_{\mathrm{pfu}}=1$	$n_{\mathrm{pfu}}=8$	$n_{\mathrm{pfu}}=1$	$n_{\mathrm{pfu}}=8$
$\tau_{\mathrm{pfu}}$	decay time of infectious virus	h	14.02	39.55	[13.87, 14.73]	[38.93, 40.22]
$V_{0}^{\mathrm{pfu}}$	initial concentration of infectious virus	$\times 10^{5}$ PFU/mL	13.64	8.30	[11.64, 15.17]	[7.46, 9.55]

**Table 4:** Parameter values obtained from fitting the Equations (3) and (4) to low and high MOI infection datasets (viral titers and total encapsulated genomes). 95% credible regions (CrRs) were constructed from MCMC fits of the model (3) and (4) to low and high MOI infection datasets.

Parameter	Description	Units	Value	95% CrR
β	rate of infection by infectious virus	$\times 10^{-6}$ mL/(PFU × h)	2.19	[0.165, 15.15]
$\tau_{E}$	length of eclipse phase	h	27	[25.94, 33.13]
$\tau_{I}$	length of infectious phase	h	30.41	[1.171, 191.07]
$p_{\mathrm{pfu}}$	infectious virus production rate	PFU/(cell × mL × h)	9.65	[8.05, 214.12]
$p_{\mathrm{rna}}$	encapsulated genome production rate	$\times 10^{4}$ RNA/(cell × mL × h)	4.11	[2.752, 99.37]
$V_{l}^{\mathrm{pfu}}\left(0\right)$	residual infectious virus in low MOI infection	$\times 10^{2}$ PFU/mL	1.80	[0.44, 4.17]
$V_{h}^{\mathrm{pfu}}\left(0\right)$	residual infectious virus in high MOI infection	$\times 10^{3}$ PFU/mL	5.12	[2.88, 9.13]

## Supplementary Materials

The following are available online at https://www.mdpi.com/1999-4915/12/5/547/s1.

## Abbreviations

The following abbreviations are used in this manuscript:

ZIKV	Zika virus
MOI	multiplicity of infection
RT-qPCR	reverse transcription quantitative polymerase chain reaction
