# Supplementary material for: Modelling Degradation and Replication Kinetics of the Zika Virus In Vitro Infection
Source: Viruses. 2020 May 15;12(5):547. doi: 10.3390/v12050547 (PMC7290367; doi:10.3390/v12050547)
Supplement: Supplementary file 1 [file viruses-12-00547-s001.pdf]

Supplementary Material

Modelling degradation and replication kinetics of Zika virus *in vitro* infection

Veronika Bernhauerová[1,2†], Veronica V. Rezelj[1], Marco Vignuzzi[1†]

[1]Viral Populations and Pathogenesis Unit, Department of Virology, Institut Pasteur, CNRS UMR 3569, F-75015, Paris, France

[2]Department of Biophysics and Physical Chemistry, Faculty of Pharmacy, Charles University, Heyrovského 1203, 500 05 Hradec Králové, Czech Republic

[†]Authors for correspondence: bernhauve@faf.cuni.cz, marco.vignuzzi@pasteur.fr

1 Parameter fitting for viral decay models

Equations (1) and (2) were numerically simulated using the Python (Python Software Foundation, Python Language Reference, version 2.7, available at https://www.python.org/) function `scipy.integrate.ode` to quantify state variables, i.e. $\log_{10} V^{\text{pfu}}$, $\log_{10} V^{\text{rna}}$ at measured time points. We fit equations (1) and (2) to the respective datasets using the Python function `scipy.optimize.least_squares` for performing unconstrained optimization on variables employing Levenberg-Marquardt method (implemented as a flag `method='lm'`). The initial concentrations of encapsulated genomes and infectious virus in the first stage, $V^{\text{rna}}(0)$ and $V_1^{\text{pfu}}(0)$, respectively, were subject to estimation. The initial concentrations of infectious virus in the remaining stages $V_{k=2,\dots,n_{\text{pfu}}}^{\text{pfu}}(0)$ were set to zero. The objective functions (SSR_{pfu}) and (SSR_{rna}) subject to minimization are given in bellow.

Alternatively, analytical solutions of (1), i.e.,

$$V^{\text{rna}}(t) = V^{\text{rna}}(0)\ \exp\left(-\frac{1}{\tau_{\text{rna}}}t\right) \tag{S1}$$

and (2), following [1], i.e.,

$$V_k^{\text{pfu}}(t) = V^{\text{pfu}}(0)\frac{\left(\frac{n_{\text{pfu}}}{\tau_{\text{pfu}}}\,t\right)^{k-1}}{(k-1)!}\ \exp\left(-\frac{n_{\text{pfu}}}{\tau_{\text{pfu}}}\,t\right) \tag{S2}$$

can be considered to evaluate the state variables and objective function, with the initial concentrations of infectious virus and encapsulated genomes, $V^{\text{rna}}(0)$ and $V^{\text{pfu}}(0)$, respectively, subject to estimation. In [2], solution of this form was proposed for the state transition model for eclipse cells at high MOI infection, assuming that all cells are infected at the beginning of an infection.

1.1 Encapsulated genomes

We fit equation (1) to experimental data described in Materials and Methods (Decay curves) by minimizing the weighted sum of squared residuals (SSR_{rna}) between the logarithm of the j-th measurement at the i-the time point $\log_{10} D_j^{\text{rna}}(t_i)$ and the respective logarithm of the solution $\log_{10} V^{\text{rna}}(t_i)$ at the time point t_i given as

$$\text{SSR}_{\text{rna}} = \sum_{i,j}\left(\frac{\log_{10} V^{\text{rna}}(t_i) - \log_{10} D_j^{\text{rna}}(t_i)}{\sigma^{\text{rna}}(t_i)}\right)^2. \tag{S3}$$

The weights were chosen to be the inverse of the sample standard deviations $\sigma^{\text{rna}}(t_i)$ of the log-experimental measures of total encapsulated genomes at each measured time t_i.

1.2 Infectious virus

We fit equations (2) to experimental data described in Materials and Methods (Decay curves) by minimizing the weighted sum of squared residuals ($\mathrm{SSR_{pfu}}$) between the logarithm of the j-th measurement at the i-th time point $\log_{10} D_j^{\mathrm{pfu}}(t_i)$ and the respective logarithm of the solution $\log_{10} \sum_{k=1}^{n_{\mathrm{pfu}}} V_k^{\mathrm{pfu}}(t_i)$ at the time point t_i given as

$$\mathrm{SSR_{pfu}} = \sum_{i,j} \left(\frac{\log_{10} \sum_{k=1}^{n_{\mathrm{pfu}}} V_k^{\mathrm{pfu}}(t_i) - \log_{10} D_j^{\mathrm{pfu}}(t_i)}{\sigma^{\mathrm{pfu}}(t_i)} \right)^2, \tag{S4}$$

The weights were chosen to be the inverse of the sample standard deviations $\sigma^{\mathrm{pfu}}(t_i)$ of the log-experimental measures of infectious virus at each measured time t_i.

2 Weibull distributed decay of infectious ZIKV

The loss of ZIKV infectivity was also modelled following the assumption that infectious virus degradation over time follows Weibull distribution, which can be mathematically expressed as [3, 7]:

$$V(t) = V_0^{\mathrm{pfu}} \exp\left[- \left(\frac{t}{\tau_{\mathrm{pfu}}} \right)^D \right] \tag{S5}$$

where τ_{pfu} (measured in (h)) is an average time for an infectious virus to lose infectivity, D is the scaling parameter and V_0^{pfu} is the initial concentration of infectious virus.

Equations (S5) were fit to experimental data described in Materials and Methods (Decay curves) as above with the objective function to be minimized given as

$$\mathrm{SSR_{pfu}} = \sum_{i,j} \left(\frac{\log_{10} V^{\mathrm{pfu}}(t_i) - \log_{10} D_j^{\mathrm{pfu}}(t_i)}{\sigma^{\mathrm{pfu}}(t_i)} \right)^2. \tag{S6}$$

The best-fit decay kinetics associated with the Weibull distribution model (S5) performed better in terms of R^2 compared to that yielded by both, the exponential and gamma distribution decay models. However, the Weibull decay model did not perform better in terms of statistical significance computed using the MCMC-accepted parameters than the gamma distribution model (equation (2) in the main text) as p-value > 0.05 (details on the calculation of the p-value are given in section 4 below). Incorporating the Weibull distributed viral decay into the model of virus-cell dynamics (3) would be difficult, because the 'age' of each infectious unit needs to be followed over time. Thus, the gamma distribution decay model was favored. Table S1 gives the best-fit values and 95% CrIs for parameters in the model (S5). The 95% credible regions and parameter posterior distributions are in Figure S2a and the associated dynamics in Figure S2b.

2.1 Parameter fitting for the main model (3)

Equations (3)+(4) were numerically simulated using the Python function `scipy.integrate.odeint` to quantify state variables, i.e. $\log_{10} V_{\mathrm{low}}^{\mathrm{pfu}}$, $\log_{10} V_{\mathrm{low}}^{\mathrm{pfu_{res}}}$, $\log_{10} V_{\mathrm{low}}^{\mathrm{rna}}$, $\log_{10} V_{\mathrm{high}}^{\mathrm{pfu}}$, $\log_{10} V_{\mathrm{high}}^{\mathrm{pfu_{res}}}$ and $\log_{10} V_{\mathrm{high}}^{\mathrm{rna}}$ over the course of infection. Fitting equations (3) to the log of experimental data $\log_{10} D_{\mathrm{low}}^{\mathrm{pfu}}$, $\log_{10} D_{\mathrm{low}}^{\mathrm{rna}}$, $\log_{10} D_{\mathrm{high}}^{\mathrm{pfu}}$ and $\log_{10} D_{\mathrm{high}}^{\mathrm{rna}}$ was performed by minimizing the weighted sum of squared residuals SSR =

$\text{SSR}^{\text{low}}_{\text{pfu}} + \text{SSR}^{\text{low}}_{\text{rna}} + \text{SSR}^{\text{high}}_{\text{pfu}} + \text{SSR}^{\text{high}}_{\text{rna}}$, where

$$\begin{aligned}
\text{SSR}^{\text{low}}_{\text{pfu}} &= \frac{1}{N^{\text{low}}_{\text{pfu}}} \sum_{i=1}^{8}\sum_{j=1}^{3} \left(\frac{\log_{10} \sum_{k=1}^{n_{\text{pfu}}} \left(V^{\text{pfu}}_{\text{k,low}}(t_i) + V^{\text{pfu}_{\text{res}}}_{\text{k, low}}(t_i) \right) - \log_{10} D^{\text{pfu}}_{j,\text{low}}(t_i)}{\sigma^{\text{pfu}}_{\text{low}}(t_i)} \right)^2, \\
\text{SSR}^{\text{low}}_{\text{rna}} &= \frac{1}{N^{\text{rna}}_{\text{low}}} \sum_{i=1}^{3}\sum_{j=1}^{3} \left(\frac{\log_{10} V^{\text{rna}}_{\text{low}}(t_i) - \log_{10} D^{\text{rna}}_{j,\text{low}}(t_i)}{\sigma^{\text{rna}}_{\text{low}}(t_i)} \right)^2, \\
\text{SSR}^{\text{high}}_{\text{pfu}} &= \frac{1}{N^{\text{high}}_{\text{pfu}}} \sum_{i=1}^{8}\sum_{j=1}^{3} \left(\frac{\log_{10} \sum_{k=1}^{n_{\text{pfu}}} \left(V^{\text{pfu}}_{\text{k,high}}(t_i) + V^{\text{pfu}_{\text{res}}}_{\text{k, high}}(t_i) \right) - \log_{10} D^{\text{pfu}}_{j,\text{high}}(t_i)}{\sigma^{\text{pfu}}_{\text{high}}(t_i)} \right)^2, \\
\text{SSR}^{\text{high}}_{\text{rna}} &= \frac{1}{N^{\text{rna}}_{\text{high}}} \sum_{i=1}^{4}\sum_{j=1}^{3} \left(\frac{\log_{10} V^{\text{rna}}_{\text{high}}(t_i) - \log_{10} D^{\text{rna}}_{j,\text{high}}(t_i)}{\sigma^{\text{rna}}_{\text{high}}(t_i)} \right)^2,
\end{aligned} \tag{S7}$$

using the Python function `scipy.optimize.least_squares` for performing optimization on variables employing Levenberg-Marquardt method (unconstrained optimization implemented as a flag `method='lm'`). The weights were chosen to be the inverse of the sample standard deviations of the log-experimental measures of infectious virus and encapsulated genomes $\sigma^{\text{pfu}}_{\text{low}}(t_i)$, $\sigma^{\text{rna}}_{\text{low}}(t_i)$, $\sigma^{\text{pfu}}_{\text{high}}(t_i)$ and $\sigma^{\text{rna}}_{\text{high}}(t_i)$ at each measured time t_i of low and high MOI dataset. We summed over the total number of measurements of infectious virus $N^{\text{pfu}}_{\text{low}}$ and $N^{\text{pfu}}_{\text{high}}$, and encapsulated genomes $N^{\text{rna}}_{\text{low}}$ and $N^{\text{rna}}_{\text{high}}$. Since we excluded data points bellow the limit of detection, we accounted for different number of measurements of infectious virus and encapsulated genomes by normalizing against the respective number of measurements $N^{\text{pfu}}_{\text{low}}$, $N^{\text{pfu}}_{\text{high}}$, $N^{\text{rna}}_{\text{low}}$ and $N^{\text{rna}}_{\text{high}}$.

3 Virus sampling for quantification

Each experimental measurement of infectious virus and encapsulated genome concentrations, V^{pfu}_k, $k = 1, \ldots, n_{\text{pfu}}$ and V^{rna}, respectively, should be reduced by 6.5% to account for the supernatant extraction at each measured time. However, the amount of virus in such a small sample is rather negligible compared to the total viral load in the supernatant and thus has only negligible impact on the overall virus dynamics. To simulate the punctual extraction of the supernatant at measured times and to show that sampling has negligible effect on the viral dynamics, we stopped the numerical integration at each time $t = 0\text{h}, 4\text{h}, 6\text{h}, 8\text{h}, 24\text{h}, 48\text{h}, 72\text{h}, 96\text{h}$ and subtract 6.5% out of the total concentration from each stage of infectious virus concentration V^{pfu}_k, $k = 1, \ldots, n_{\text{pfu}}$ and encapsulated genome concentrations V^{rna} and re-initiate the simulation with these reduced values as new initial conditions. This routine is repeated at every measured time point. We show the best-fit solution of the model (3)+(4) (best-fit parameter values are in Table 4) with and without sampling adjustment in Figure S3.

4 MCMC computations and statistical analysis

To infer posterior parameter distributions, we employed a Python module `emcee` [4], which is an implantation of Goodman and Weare's Affine Invariant Markov chain Monte Carlo (MCMC) Ensemble sampler [5]. Twenty walkers were log-uniformly distributed within the close proximity of the best-fit parameter set to perform MCMC inference. A proposed step $\vec{x}$ was accepted or rejected with the acceptance probability $\exp^{-0.5 \times SSR(\vec{x})}$ (as in [6]), where $SSR(\vec{x})$ is the weightned sum of squared residuals between the solution of the model and experimental measurements. For viral decay models (1), (2) and (S5) we implemented a burn-in of 200 steps for each MCMC run. Another 1,000 steps were run, thus totalling in 20,000 parameter sets that were used to generate the posterior parameter

distributions.

In the case of the main model (3)+(4), we ran the MCMC process for 60,000 steps, totalling in 1,200,000 parameter sets. The convergence of the MCMC samples was graphically inspected. Due to computational limitations, we performed thinning to reduce autocorrelation in MCMC chains and kept every tenth parameter set for each chain. The autocorrelation function (AFC_k), calculated as

$$AFC_k = \frac{s_k}{s_0}, k \geq 0, \tag{S8}$$

where

$$s_k = \frac{1}{n} \sum_{i=k+1}^{n} (y_i - \bar{y})(y_{i-k} - \bar{y}), \tag{S9}$$

and $\bar{y}$ and s_0 are the mean and variance of the time series $y_1, \ldots, y_n$, respectively, was plotted to asses the correlation between the samples k steps apart (Figure S4, lag on x-axis). The Figure S4a shows values of the lag-k ACF against increasing values of k for unthinned chains. The autocorrelation values drop slowly for larger k for the parameters β, τ_E, τ_I, p_{pfu} compared to the parameters $V_h^{\text{pfu}}(0)$ and $V_h^{\text{rna}}(0)$. Autocorrelation after thinning on Markov chains is displayed in Figure S4b.

Trace plots in Figure S5 show the sampled values of the model parameters over time. This plot helps to judge how rapidly the MCMC process converges to marginal parameter posterior distribution. For the parameters β, τ_E, τ_I, p_{pfu} and p_{rna}, the chains seem well burnt after approximately 2500 steps. We thus set the burn-in to double, i.e. 5000 steps. The thinned samples, after the burn-in was discarted, were used to generate the posterior parameter distributions in Figure 5 the main text.

Statistical significance was quantified using a bootstrap t-test. To determine whether two mathematical models of viral decay are statistically different (one model performs better then the other), we calculated the Akaike Information Criterion for small sample size AIC_C as

$$AIC_C = n \log\left(\frac{SSR}{n}\right) + 2\,k + 2\,k\,\frac{k+1}{n-k-1}. \tag{S10}$$

We then sampled (with replacement) 1000 parameter sets out of the total of 20000 parameter sets obtained from MCMC simulation for each viral decay model and calculated the fraction of times the AIC_C of one model was smaller than that of the other. We repeated the procedure one hundred times and calculated the final p-value as the mean of all bootstrap p-values.

References

[1] Hurtado PJ, Kirosingh AS. 2019. Generalizations of the 'Linear Chain Trick': incorporating more flexible dwell time distributions into mean field ODE models. Journal of Mathematical Biology 79:1831–1883.

[2] Kakizoe Y, Nakaoka S, Beauchemin CA, Morita S, Mori H, Igarashi T, Aihara K, Miura T, Iwami S. 2015. A method to determine the duration of the eclipse phase for in vitro infection with a highly pathogenic SHIV strain. Scientific Reports. 5:10371.

[3] Okagbue HI, Adamu MO, Anake TA. Ordinary Differential Equations of the Probability Functions of the Weibull Distribution and their Application in Ecology. 2017. Journal of Economic Literature (JEL).

[4] Foreman-Mackey D, Hogg DW, Lang, D and Goodman J. 2013. emcee: the MCMC hammer. Publications of the Astronomical Society of the Pacific 125:306–312.

[5] Goodman J and Weare J. 2010. Ensemble samplers with affine invariance. Communications in applied mathematics and computational science 5:65–80.

[6] Paradis EG, Pinilla LT, Holder BP, Abed Y, Boivin G, Beauchemin CA. 2015. Impact of the H275Y and I223V mutations in the neuraminidase of the 2009 pandemic influenza virus in vitro and evaluating experimental reproducibility. PLoS One 10(5):e0126115.

[7] Beauchemin CAA, Kim YI, YuQ, Ciaramella G, DeVincenzo JP. 2019. Uncovering critical properties of the human respiratory syncytial virus by combining in vitro assays and in silico analyses. PLoS One 14:e0214708.

Figures

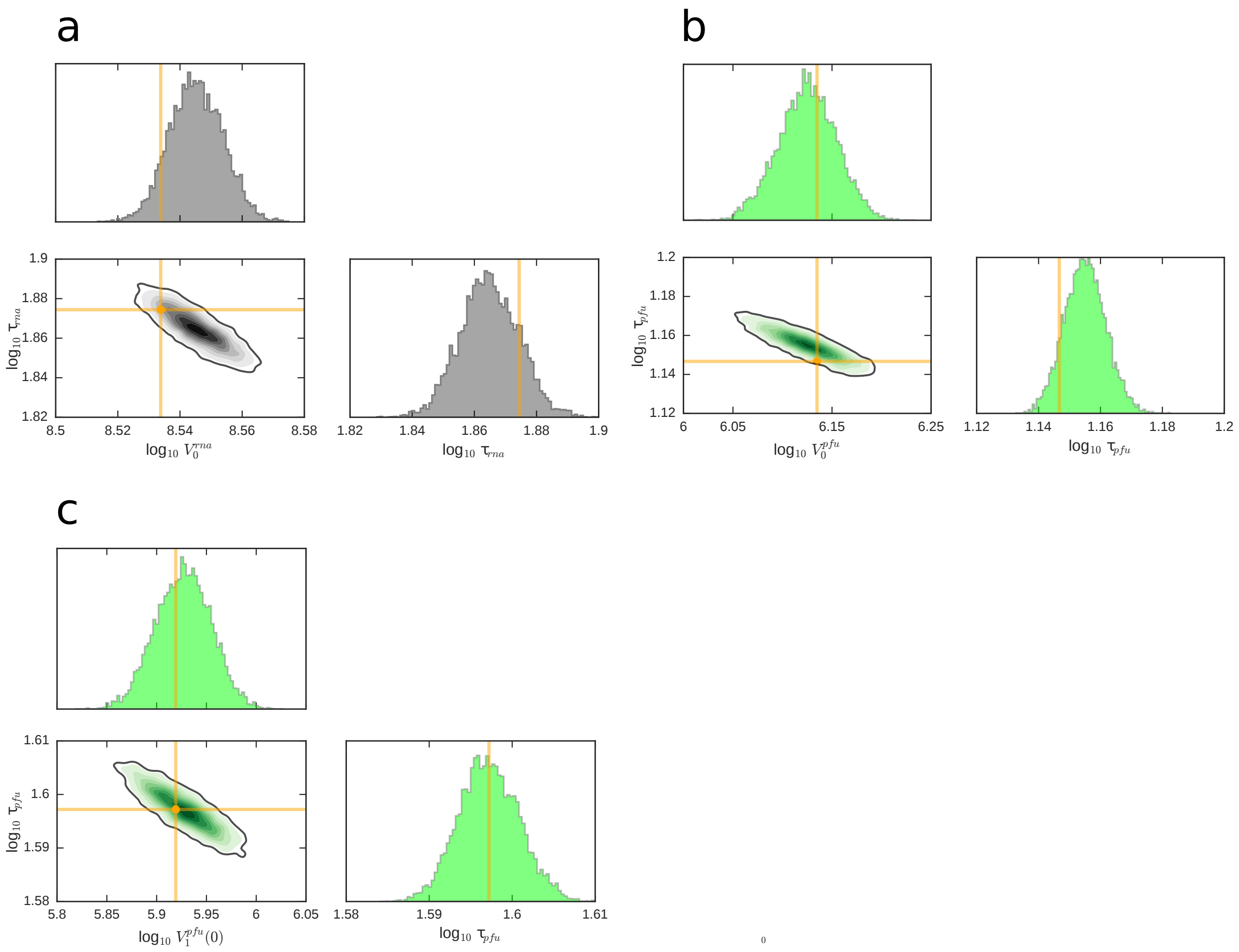

Figure S1. Parameter posterior distributions and pair-wise posterior plots obtained from MCMC run of the decay model for **(a)** encapsulated genomes (equation (1)) and **(b-c)** infectious virus (equations (2)), assuming **(b)** exponentially distributed decay time ($n_{\text{pfu}} = 1$) and **(c)** gamma distributed decay time ($n_{\text{pfu}} = 8$). The orange targets indicate the best-fit parameter values given in Tables **(a)** 2 and **(b-c)** 3. The solid dark lines enclose the 95% credible regions.

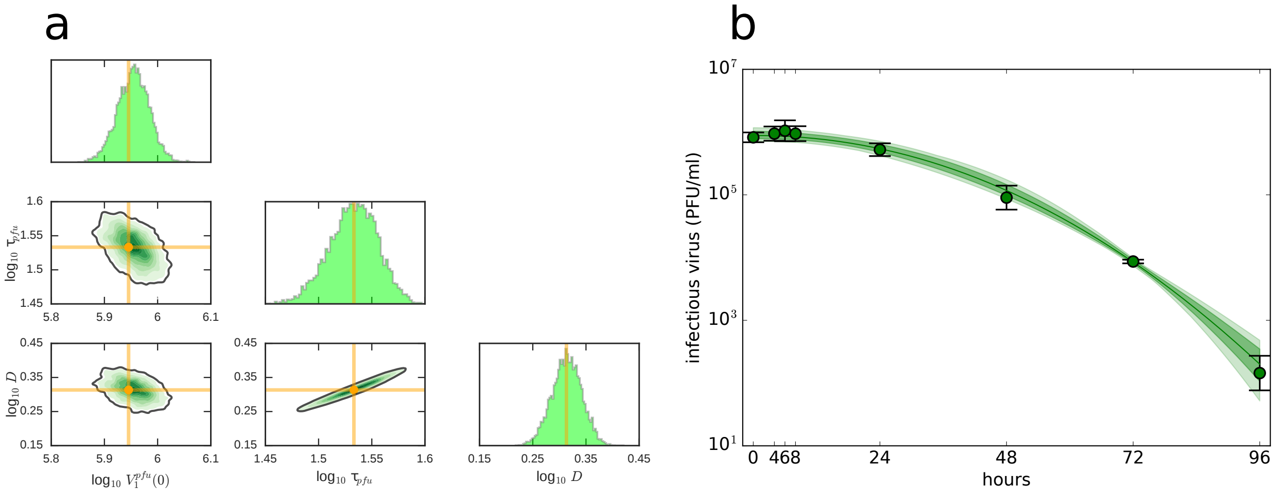

Figure S2. **(a)** Parameter posterior distributions and pair-wise posterior plots obtained from MCMC run of the Weibull decay model (S5). The solid dark lines enclose the 95% credible regions. **(b)** The best-fit of the model (S5) is displayed as a solid green line. The light shading around the best-fit corresponds to the model kinetics associated with MCMC-accepted parameters. The dark shading represents 95% credible region. Data are shown as the mean ± standard deviation.

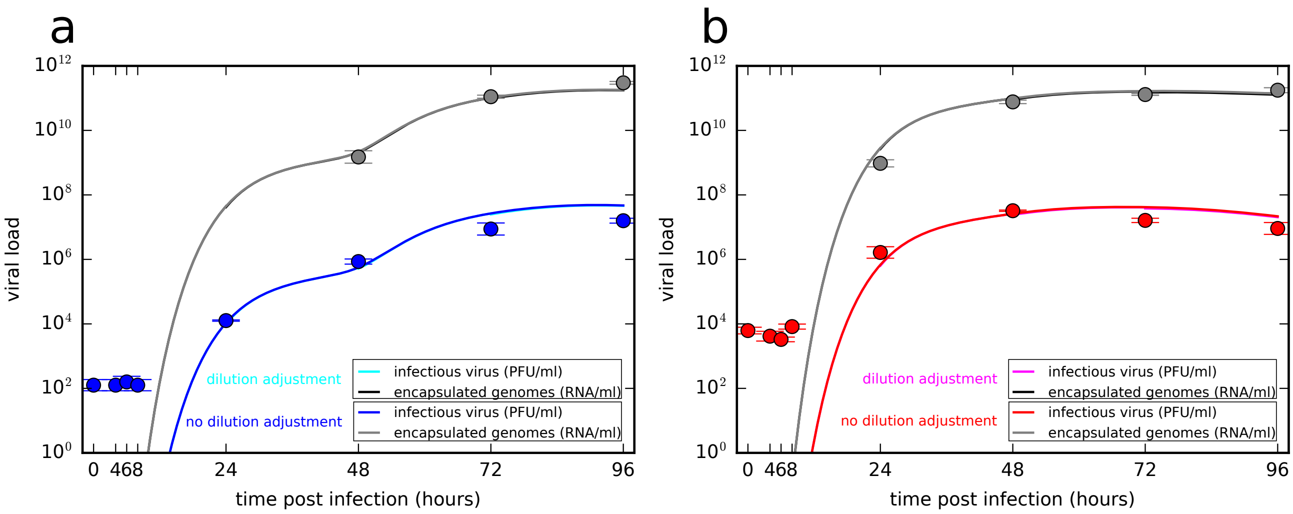

Figure S3. Simulated time course dynamics of infectious virus and encapsulated genomes yielded by the model (3)+(4) using the best-fit parameters in Table 4 and taking timely extractions of the supernatant into account. **(a)** low MOI infection dynamics, **(b)** high MOI infection dynamics. In both figures, 'no dilution adjustment' refers to the continuous simulation whereas 'dilution adjustment' refers to the sequentially restarted simulation where we adjust for removal of the supernatant for quantification.

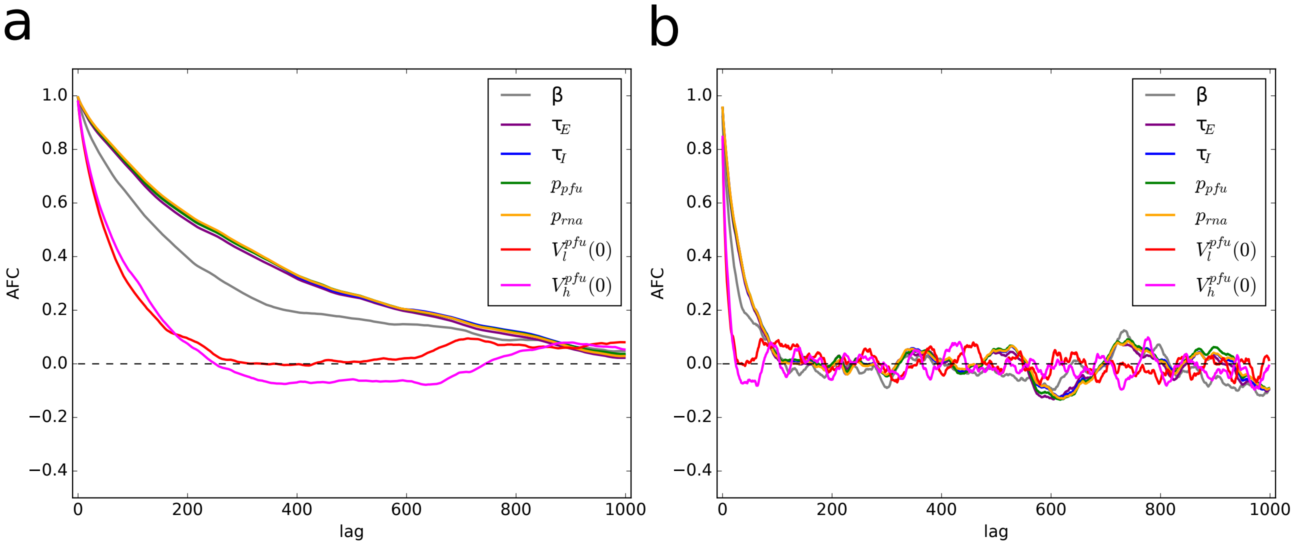

Figure S4. Graphical diagnostics of the MCMC run. Autocorrelation of the parameters as a function of the sample lag in one of the **(a)** unthinned and **(b)** thinned Markov chains. Thinning was performed using every tenth parameter set in each chain to reduce autocorrelation.

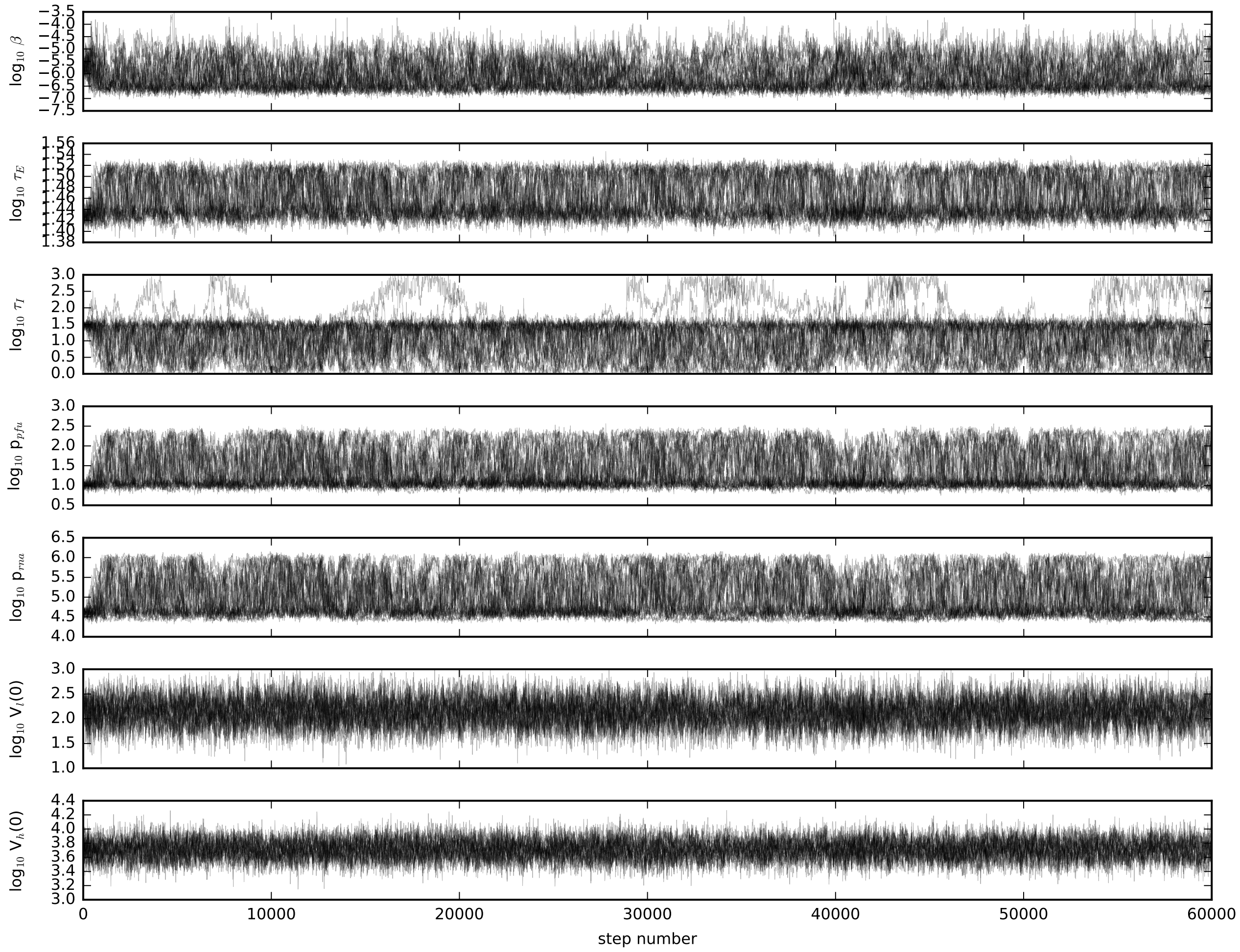

Figure S5. Trace plots of the thinned MCMC chains.

Tables

Table S1. Parameter values obtained from fitting equations (S5) to infectious virus decay data and 95% CrRs were constructed from the MCMC fits of the model (S5).

parameter	description	value	95% CrR
τ_{pfu} (h)	decay time of infectious virus	34.14	[30.60, 37.57]
D (dimension-less)	scaling constant	2.06	[1.81, 2.33]
V_0^{pfu} ($\times 10^5$ PFU/ml)	initial infectious virus	8.82	[7.85, 10.28]
